# Associations between Wastewater Microbiome and Population Smoking Rate Identified Using Wastewater-Based Epidemiology

**DOI:** 10.1021/envhealth.3c00105

**Published:** 2023-10-19

**Authors:** Jiangping Wu, Shuxin Zhang, Yan Chen, Jiawei Zhao, Tanjila Prosun, Jake William O’Brien, Jochen F. Mueller, Ben J. Tscharke, Lachlan J.M Coin, Stephen P. Luby, Faisal I. Hai, Tanya Buchanan, Guangming Jiang

**Affiliations:** † School of Civil, Mining, Environmental and Architectural Engineering, University of Wollongong, Wollongong 2522, Australia; ‡ Queensland Alliance for Environmental Health Sciences (QAEHS), The University of Queensland, Brisbane 4102, Australia; § Department of Clinical Pathology, The University of Melbourne, Parkville 3052, Australia; ∥ Department of Microbiology and Immunology, The University of Melbourne, Parkville 3052, Australia; ⊥ Division of Infectious Diseases and Geographic Medicine, Stanford University, Palo Alto, California 94034, United States; # Cancer Council Australia, Sydney 2000, Australia; ∇ School of Health and Society, University of Wollongong, Wollongong 2522, Australia

**Keywords:** Smoking, Wastewater, Metagenomics, Wastewater-based epidemiology, Microbial biomarker, Human gut

## Abstract

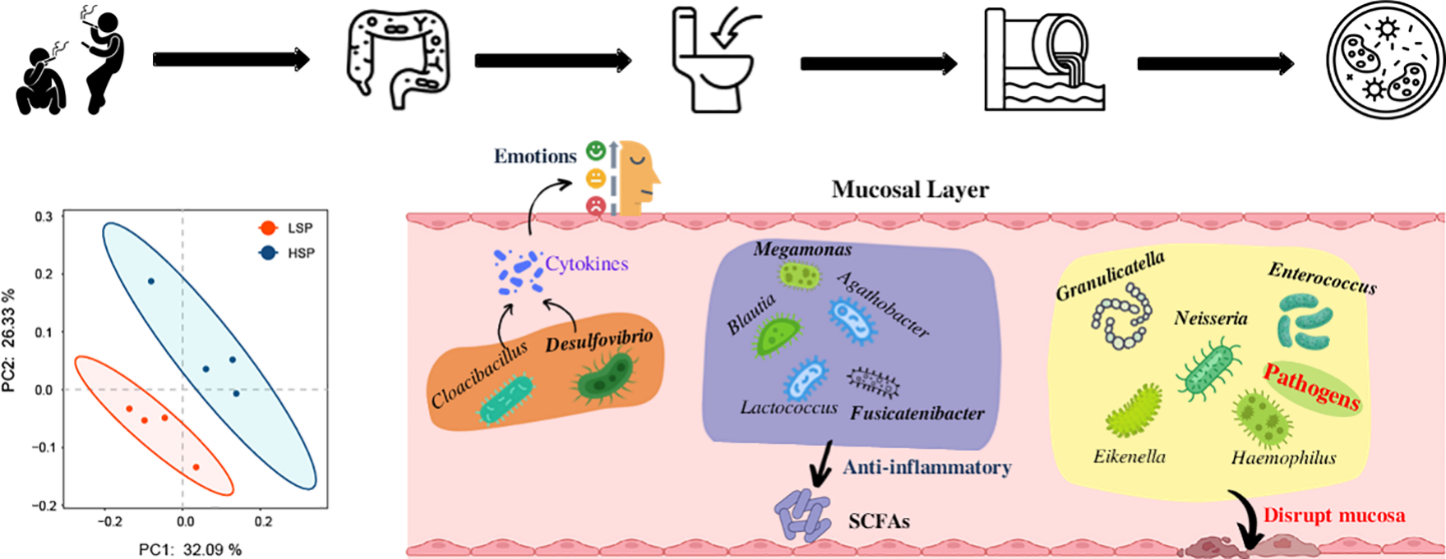

Tobacco use is known to cause health damage, partly by changing the mouth, respiratory tract, and gut-related microbiomes. This study aims to identify the associations between the human microbiome detected in domestic wastewater and the population smoking rate. Metagenomic sequencing and a biomarker discovery algorithm were employed to identify microorganisms as potential microbial biomarkers of smoking through wastewater-based epidemiology. Wastewater samples were collected from selected catchments with low and high smoking rates, i.e., 11.2 ± 1.5% and 17.0 ± 1.6%, respectively. Using the linear discriminant analysis effect size (LEfSe) method, *Neisseria*, *Desulfovibrio*, *Megamonas*, *Blautia*, *Fusicatenibacter*, *Granulicatella* and *Enterococcus* were suggested as potential biomarker microorganisms. A higher abundance of pathogens, including *Neisseria*, *Eikenella* and *Haemophilus*, was associated with the high smoking rate, likely because of their colonization in smoking-disturbed human guts. The identified potential microbial biomarkers reflect the change of the human gut microbiome due to the long-term smoking behavior. The metagenomic analysis also indicates that smoking upregulates microbial gene expression of genetic information processing, environmental information processing, and cell wall peptidoglycan cleavage, while it downregulates amino acid, lipid, and galactose metabolisms. The findings demonstrate the potential of microbial biomarkers for the surveillance of smoking through a wastewater-based epidemiology approach.

## 1 Introduction

Smoking is one of the major public health threats globally. More than 60 known cancer-causing chemicals are found in tobacco smoke, which kills over 8 million people among about 2 billion tobacco users annually.^1,2^ In Australia, smoking is the leading cause of cancer (44% of the cancer burden) and the most important preventable cause of illness and death, with the social cost estimated at $136.9 billion in 2015–2016.^3^ It is thus important to monitor tobacco use in the population for public health management.

Wastewater-based epidemiology (WBE) has been used to estimate tobacco use in a specific population by analyzing the presence of biomarker compounds such as nicotine, cotinine, and hydroxycotinine in wastewater.^4,5^ This approach provides an estimate of tobacco use in a population, as it captures data from all members of the catchment, including those who may not participate in traditional surveys or self-report their behavior. Since nicotine has also been used as a smoking cessation aid, anabasine and anatabine were recommended as tobacco-specific biomarkers in urine.^6^ However, the degradation of these biomarker compounds in wastewater, especially their deconjugation process, compromised their use as a stable biomarker of smoking.^4,5^ It is thus essential to search for new alternate wastewater biomarkers for the population smoking rate.

The influent wastewater of wastewater treatment plants (WWTPs) usually provides a good reflection of the human microbiome in sewer catchments.^7^ Based on 16S rRNA gene sequence data, domestic wastewater was found to represent the fecal microbial community of human populations, and it captures population-level traits such as obesity.^8^ Many studies have reported that smoking raises intestinal pH and thus disturbs the gut microbiome.^9^ Also, toxic smoking substances reduce endogenous antioxidants, alter intestinal mucins, and tight junction proteins.^10^ Smoking was also associated with the colonization of bacteria that causes acute infectious diseases or chronic diseases including cancer.^11^ Many studies have reported the alterations of microbiomes in human or animal oral, airway, and gut environments. For example, a study in a Bangladeshi population found a consistent association between smoking status and quantity with microbial community changes in the gut.^12^ These changes in the human microbiomes due to smoking potentially can be reflected in the wastewater microbiome, which is a collective sample of the population in a connected sewer catchment.

Metagenomic sequencing of wastewater samples can provide much more information about the whole microbiome than other detection methods such as 16S amplicon sequencing or qPCR.^13^ The comprehensive metagenomics-based approach was adopted for the global surveillance of antibiotic resistance in wastewater.^14,15^ During COVID-19, specific microbial species detected by metagenomics in wastewater showed a potential association with SARS-CoV-2 infection.^16^ Essentially, metagenomics of wastewater was demonstrated to be a surveillance tool offering extensive microbiome data for antibiotic resistance, public health and infectious diseases.^14,17^ It is thus adopted as a tool to delineate the associations of the microbiome with the population smoking rate using a WBE approach.

Recent metagenomic studies of human microbial communities identified biomarkers for lifestyle and diseases, using different biomarker discovery algorithms.^18^ The linear discriminant analysis (LDA) effect size (LEfSe) method is the most widely used algorithm that can support high-dimensional metagenomic data of wastewater. It determines the biomarkers, such as organisms, genes, or functions, that are most likely to explain differences caused by lifestyle or diseases by the combination of statistical significance, biological consistency, and effect relevance.^19^

This study aims to discover potential microbial biomarkers that are associated with the population smoking rate by analyzing wastewater samples collected from catchments with varying levels of smoking during the census period in Australia. The approach includes the analysis of wastewater microbiomes using Nanopore metagenomic sequencing, identification of microorganisms differentiating smoking rates through the well-established LEfSe biomarker discovery algorithm, cross-validation with chemical marker compounds of smoking, and functional annotations of the metagenome for the further validation of potential biomarkers against the reported impacts of smoking on gut microbiomes. The results of this study are expected to support the search for new and alternative microbiome-based biomarkers for the further development of the WBE for smoking.

## 2 Materials and Methods

### 2.1 Wastewater Sampling

Wastewater samples were collected as a part of the SewAus wastewater sampling program which was conducted on the Australian Census day.^20^ Briefly, 24-h composite wastewater influent samples were collected using on-site refrigerated autosamplers at each WWTP operating in the most representative mode available at each site.^20^ The eight WWTPs selected for this study cover capital and regional areas across Queensland (QLD) and New South Wales (NSW) in Australia. The population of each WWTP ranged from 70,000 to 1.3 million (Table S1).

Wastewater samples were aliquoted into precleaned and sterilized polyethylene terephthalate bottles and preserved in 20% glycerol or 2 M hydrochloric acid for sequencing or nicotine analysis, respectively. Both were frozen onsite before being transported frozen to the laboratory. Samples were stored at −20 °C or −80 °C before the sequencing or nicotine analysis, respectively. Chemical analyses were conducted as a part of the National Wastewater Drug Monitoring Program as per the methodology described by O’Brien et al.^21^ The nicotine daily loading rate was calculated based on the nicotine concentration, WWTP daily flow rate, and catchment population.

Data of the population smoking rate (percentage of people aged 18 years and over who were current smokers) were published by Australian Bureau of Statistics from the National Health Survey.^22,23^ The percentage of smokers for catchment areas covered by a WWTP was calculated based on this published data and the population for the service areas. The WWTPs were selected because their population smoking rate falls into two distinct ranges: low smoker percentage (LSP) catchments (11.2 ± 1.5%, i.e., WWTPs 1–4) and high smoker percentage (HSP) catchments (17.0 ± 1.6%, i.e., WWTPs 5–8).

### 2.2 Nanopore Library Preparation and Sequencing

One influent wastewater sample from each WWTP was homogenized with vigorous shaking, and 50 mL was filtered using 0.22 μm mixed cellulose ester filters (Sigma Millipore) and stored at −80 °C before DNA extraction. DNA was extracted from each sample following the manufacturer’s instructions (MP Biomedicals, U.S.A.) for FastDNA SPIN Kits for soil. The DNA concentration was measured by a Qubit 4 Fluorometer with a Quant-iT PicoGreen dsDNA Assay Kit (Thermo Fisher, U.S.A.).

NEBNext End repair/dA-tailing Module (New England BioLabs, 7546) and NEBNext FFPE DNA Repair Mix (New England BioLabs, 6630) were used to convert the extracted DNA fragments to repaired DNA with 5′-phosphorylated, 3′-dA-tailed ends. The library was then prepared using an Oxford Nanopore Technologies (ONT) Ligation library preparation kit (SQK-LSK-109, ONT). 200 ng of DNA per sequencing run was loaded onto R9.4 flow cells with a last volume of 75 μL. The ONT GridION sequencer was used to sequence prepared libraries, one flow cell for each sample, over 72 h.

### 2.3 Metagenomic Data Analysis

Genomic reads in Fastq files were filtered with a quality score of >9 following the Nanopore sequencing. The long-read metagenomic data was assembled with Flye (v.2.9.1-b1780) using the parameter “-meta”.^24^ The reads were then aligned against the assembled genome using minimap2 (v.2.24-r1122).^25^ Racon was used to polish and correct the aligned results by repeating the process three times.^26^

Prodigal (v.2.6.3) was then utilized for open reading frame (ORF) prediction following the correction with a range of output files including ORF nucleic acid sequence, amino acid sequence, gff, etc.^27^ The parameter “-p meta” was used with Prodigal for the genes prediction based on the wastewater metagenomes.^28^ The genes were then clustered using Cd-hit with a similarity threshold of 95%. The relative abundance of gene expression was normalized in transcripts per million (TPM) by Salmon (v.1.8.0).^29^ Missing values are filled in with zeros.

Microbial community was identified by searching the clustered proteins in the Unified Human Gastrointestinal Genome (UHGG) (version 2.0) database using Blastp and an E-value threshold of 1 × 10^–10^.^30^ The microorganisms screened by the UHGG database will be used for subsequent microbiome analysis.^31^ Eggnog-mapper (v.2.1.7) was used to implement function annotations of metagenomic reads for COG (Cluster of Orthologous Groups), CAZy (Carbohydrate-Active Enzymes), and KEGG (Kyoto Encyclopedia of Genes and Genomes).^32^

### 2.4 Analysis of Microbial Community Diversity

To analyze the diversity of human fecal microbial community in wastewater, the alpha diversity (Shannon index and Simpson index) was calculated with vegan package in *R*.^33^ Alpha diversity describes the diversity within a community, including the richness, evenness and phylogenetic diversity.^34^ To evaluate differences in species complexity among samples, beta diversity analysis was conducted in combination with dimensional reduction methods. The results were generated using a permutation multivariate analysis of variance (PERMANOVA). This is an F-statistic-based ANOVA of the relative abundance, which calculates the Bray–Curtis distance between samples. The Adonis analysis was performed using adonis2 in the vegan *R* package.

### 2.5 Identifying Associations between Metagenomic Microbiome and Smoking Rate

The associations between the metagenomic microbiome and smoking rate were determined through the identification of potential marker microorganisms for smoking, based on the relative abundance data of the human gut microbiome in wastewater. The Spearman correlation coefficients were calculated between the relative abundance of microorganisms and the population smoking rate in order to investigate their associations and its significance (statistically significant if *p* < 0.05).^35^ The variance was calculated for the microorganisms screened by correlation coefficients. The calculations were performed with the “psych” and ggplot2 packages in *R*. GraphPad Prism 5.0 was used to conduct the Mann–Whitney tests to evaluate whether there were significant differences in the bacteria’s relative abundance.

Significantly associated microorganisms (*p* < 0.05) based on the correlation coefficients were then used in the LEfSe analysis.^19^ This analysis was performed on the ImageGP Platform.^37^ Following the discovery of potential microbial biomarkers, Pearson correlation analysis was conducted to examine the relationship between daily nicotine loading rate and microbial communities across WWTPs. Subsequently, the top 10 microorganisms with the highest differential relative abundance (*p* < 0.05) were selected to validate the potential biomarkers.

The symbiotic relationship of potential microbial biomarkers with other microorganisms was revealed by the co-occurrence correlation network. Global Pearson correlations were calculated. The co-occurrence network of associations between pairs of genus with *p*-values < 0.01 and |*r*| > 0.6 was plotted in Gephi (version 0.10.1).^38^

Spearman correlation analysis was also performed between the COG, KEGG data sets and smoking rate, to identify COGs and KOs (functional ortholog) that are significantly associated (*p* < 0.05) with smoking. The identified data sets (CAZy data, COG classification data and KEGG L1 and L2 data) were further analyzed through LEfSe analysis to uncover associations of COGs and KOs to smoking.

## 3 Results and Discussion

### 3.1 Human-Related Microbial Composition in Wastewater

**Figure 1:**
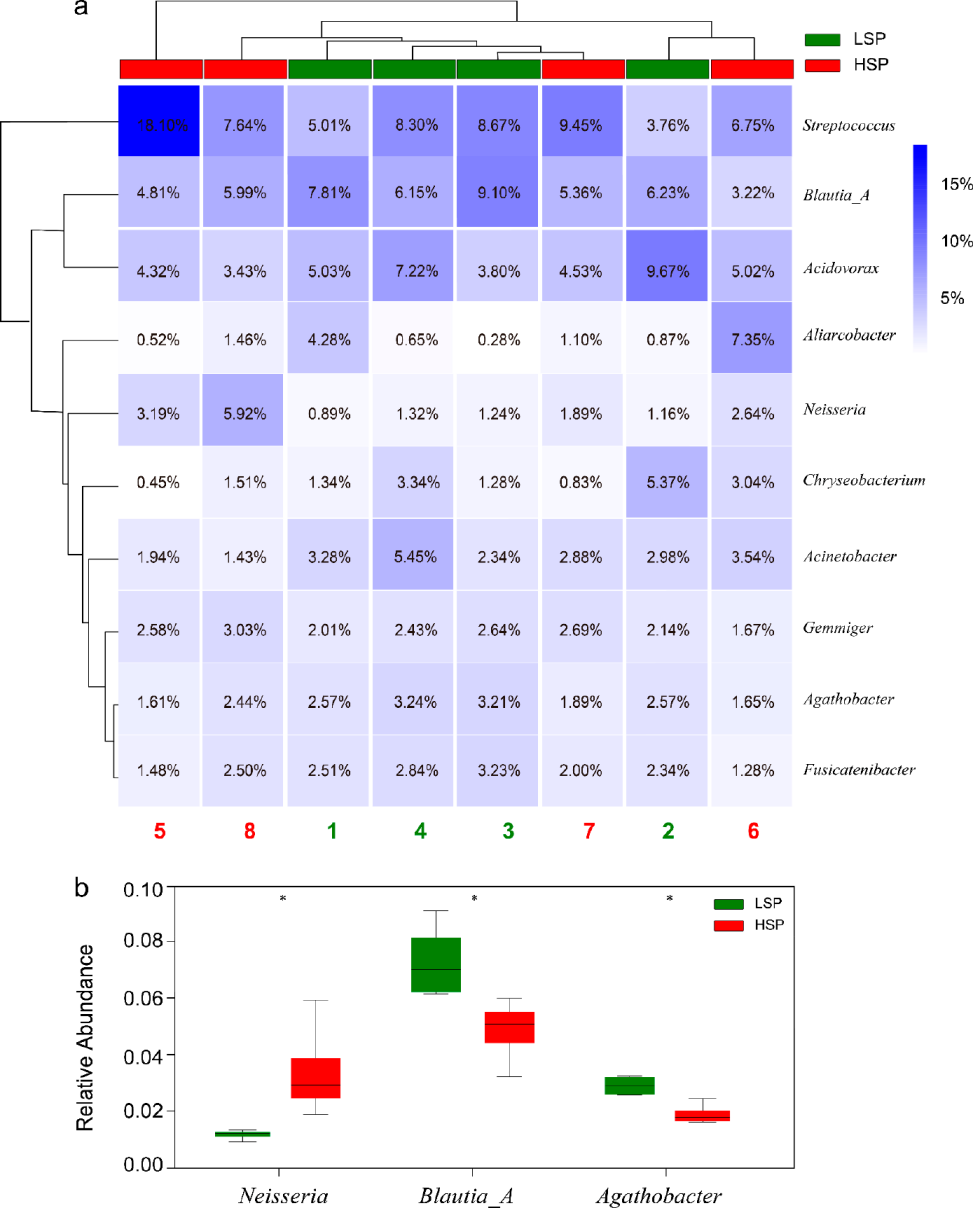
(a) Top 10 most abundant genera of the human gut microbial community detected in wastewater. (b) Box plots showing the relative abundances of *Neisseria*, *Blautia_A* and *Agathobacter*, respectively, with significant differences (*p* < 0.05, indicated by *) between low (LSP) and high (HSP) smoker percentage in the population.

An average of 11.9 GB (9.0–19.0 GB) of data per wastewater sample was obtained using Nanopore high-throughput sequencing, with the N50 at around 2.5 kb. The sequencing depth of all wastewater samples is sufficient to cover the richness of microbial diversity in the entire sample (Figure S1). Figure 1 shows the microbial composition of the human gut microbiome (filtered by the UHGG database) detected in wastewater at the genus level. Firmicutes, Proteobacteria, Bacteroidota and Actinobacteriota account for about 90% of the total abundance (at the phyla level) (Figure S2). The primary microbial genera are *Aliarcobacter*, *Chryseobacterium*, *Fusicatenibacter*, *Neisseria*, *Agathobacter*, *Gemmiger*, *Acinetobacter*, *Acidovorax*, *Blautia_A* and *Streptococcus*. Collectively, these genera constitute approximately 40% of the microbial community. Notably, the microorganisms present at relatively higher levels were predominantly human gut-associated microorganisms, which is in line with previous observations.^39,40^

Among them, there were significant differences (*p* < 0.05) in the relative abundances of *Neisseria*, *Blautia_A* and *Agathobacter* (Figure 1b). *Neisseria* is a commensal pathogen and an important risk factor for meningococcal disease, whose presence in the human nasopharynx was reported to be increased by smoking or exposure to smoke.^41^*Blautia* reduces inflammation and maintains intestinal microecology, and its abundance was observed to decrease with environmental tobacco smoke exposure in children.^42^*Agathobacter*, a producer of butyrate, whose abundance was reported to decrease in the esophageal squamous cancer disease group.^43^ These results of human gut microbiome in wastewater are largely consistent with previous observations directly in the human gut environment, which supports further analysis to identify potential biomarker microorganisms for WBE.

**Figure 2:**
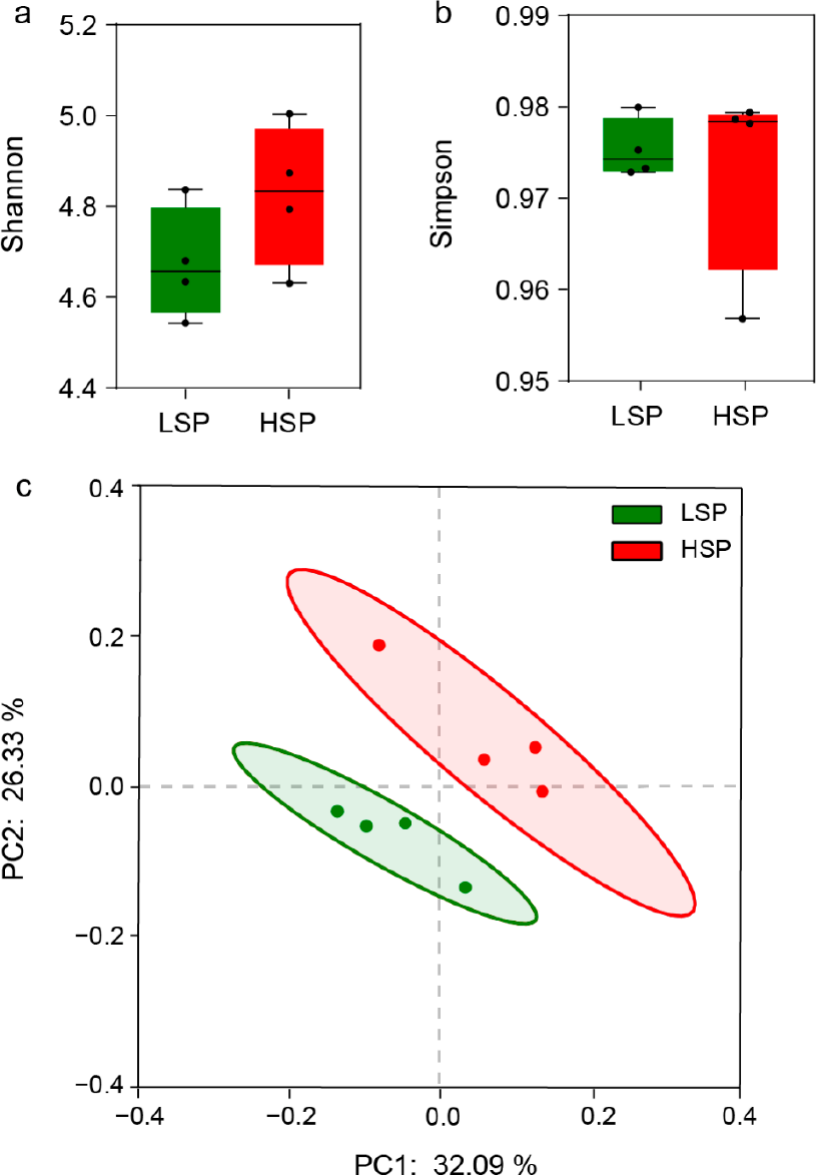
(a) Shannon index, (b) Simpson index, and (c) PCoA using the Bray–Curtis metric show diversity differences in microbial community of LSP and HSP samples.

The human gut microbiome in wastewater showed no significant difference of the α diversity in terms of Shannon index and Simpson index (Figure 2a and b, respectively) between the two groups, i.e., LSP and HSP. This indicates that different smoking rates did not cause a significant variation in the richness, evenness, and phylogenetic diversity. The Shannon Index and Simpson Index are similar to the reported diversity of human gut flora.^44^ For the β diversity of microorganisms, the principal coordinates analysis (PCoA) based on Bray–Curtis distance matrices (Figure 2c) shows a clear separation between the LSP and HSP groups. The PERMANOVA analysis also indicated a significant difference (*p* = 0.041). The smoker percentage in the population explained up to 28% of the diversity differences between the two groups. This is likely due to the fact that smoking can cause significant variances to the human intestinal microbes.^45^ This observation of no changes in α diversity, but significant changes in β diversity, of the human gut microbiome in wastewater because of smoking is similar to microbial diversity changes observed in human intestinal microbiota.^46^

### 3.2 Microbial Biomarker Discovery by LEfSe

A total of 261 human gut bacterial genera detected in wastewater were screened by Spearman’s correlation coefficient for the correlation of their relative abundances with the population smoker percentage (*p* < 0.05). The top 10 genera with the highest absolute correlation coefficients are shown in Figure S3. The human gut microorganisms correlated with the population smoking rate were mainly *Firmicutes* and *Actinobacteriota*.

**Figure 3:**
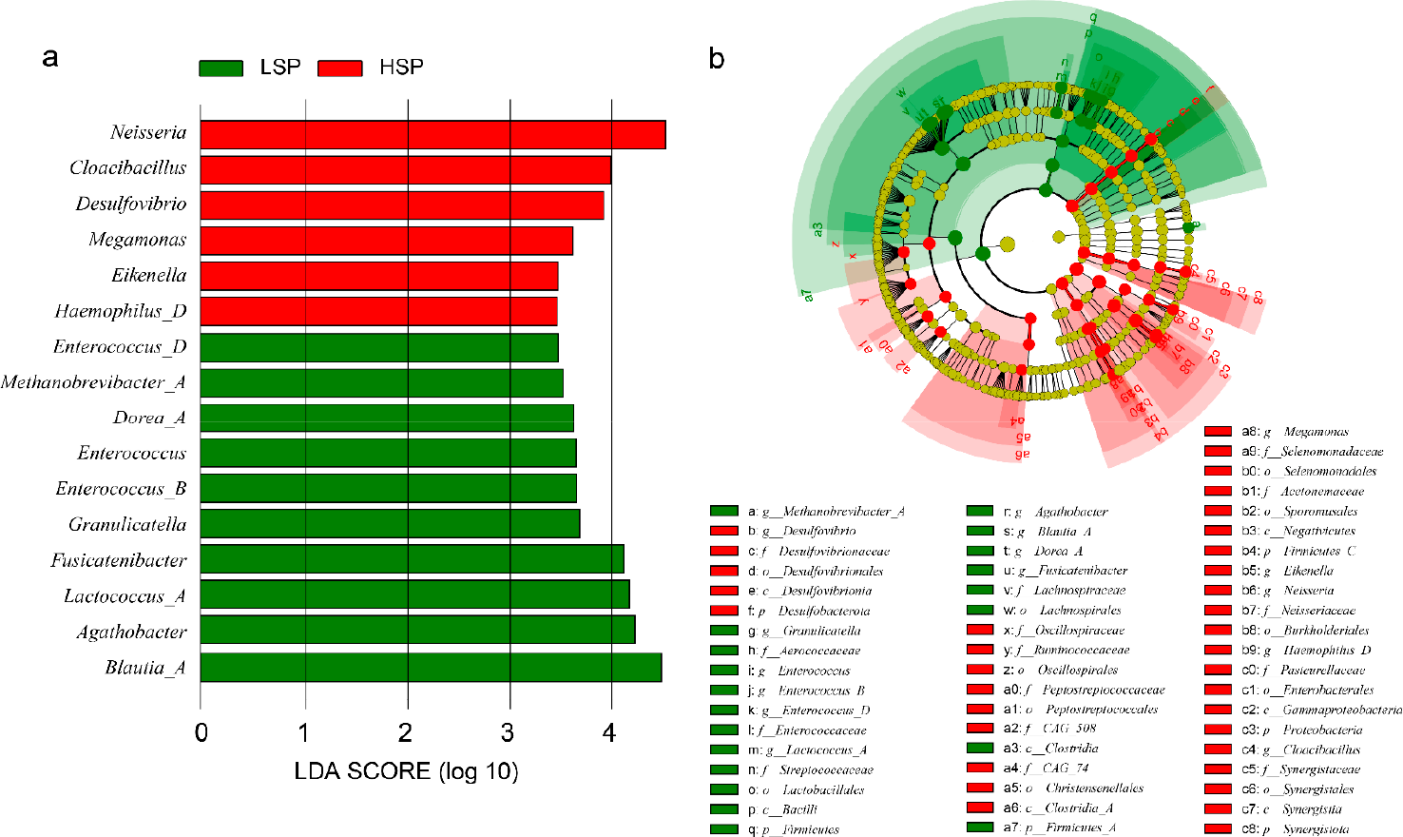
Screening of human gut microbiome in wastewater to discover potential marker microorganisms for the population smoking rate. (a) LEfSe analysis suggested 16 microbial biomarkers at the genus level (*p* < 0.05, LDA ≥ 3.5). (b) Phylogenetic dendrogram of biomarker bacteria suggested by LEfSe.

Using LEfSe for the biomarker discovery with the criterion of LDA ≥ 3.5 and *p* < 0.05, 16 microbial genera were suggested (Figure 3a and b). Among them, *Neisseria* had the highest LDA score and a high correlation with the daily loading rate of nicotine as a chemical biomarker (*r* = 0.81). *Neisseria* can colonize and invade hosts through the expression of surface adhesion proteins and can penetrate mucous membranes of human guts to cause various forms of disease and a high lethality rate.^47^*Cloacibacillus* utilizes mucin as its sole carbon source, which is linked to various cancers, cellular signaling pathways, and the blood–brain barrier.^48,49^ It was reported to be elevated in epileptic patients and has been shown to be involved in mood regulation.^50^ Also, higher levels of *Cloacibacillus porcorum* were discovered in the mouths of individuals who smoke traditional cigarettes compared to never smokers.^51^ Nicotine is a potent alkaloid that acts on nicotinic acetylcholine receptors, producing a sense of pleasure, regulating mood, and thus producing addiction. This is reflected by the increased level of *Cloacibacillus*, which is also known to be involved in mood regulation.^50^

For the third biomarker, *Desulfovibrio*, its abundance in the gastrointestinal tract of individuals who smoke was reported to be increased.^52^*Desulfovibrio* produces hydrogen sulfide and lipopolysaccharide, which can trigger alpha-synuclein accumulation, potentially contributing to Parkinson’s disease.^53^ Another biomarker, *Megamonas*, was reported to be involved in the fermentation of glucose to produce propionic and butyric acids, and its levels were elevated in the gut microbiome of smokers and those with higher BMI, correlating with the inflammatory phenotype of the host.^54^

Some marker microorganisms, such as *Cloacibacillus*, *Eikenella* and *Haemophilus*, are pathogens or associated with cancers.^55−57^ Their association with a high population smoking rate is likely due to the exacerbated risk of respiratory infections and other invasive infections such as bacteremia and meningitis caused by smoking. Nicotine and other tobacco metabolites can be present in the human gastrointestinal tract, affecting its secretions and metabolism and the intestinal microbiome. For instance, nicotine relaxes smooth muscle and increases pepsin secretion.^58^ Previous studies have shown that smoking generates reactive oxygen species (ROS) in the blood, leading to oxidative stress and activation of JNK and p38 signaling.^59^ Smoking also alters the mucin composition of the gut and cellular tight junction molecules, in addition to causing local acid–base imbalances in the gut.^60^

Smokers tend to have mucosal immunosuppression, which can make the host more susceptible to certain pathogenic bacteria and increase the risk of various infections.^52^ The suppressed mucosal layer of the intestine and the altered intestinal environment of smokers make it easier for pathogenic bacteria to invade and colonize. *Neisseria* is well adapted to the mucosal surface of the intestine for colonization and crosses the blood–brain barrier to cause hematogenic diseases. Pathogenic bacteria are more likely to penetrate the mucosa and enter the bloodstream, causing systemic diseases such as bacteremia and sepsis among smokers. The identified marker microorganisms for the high smoking rate are mostly hematogenic bacteria.

Biomarkers associated with low smoking rate, including *Blautia*, *Lactococcus, Fusicatenibacter* and *Agathobacter*, produce butyric and acetic acid, which have important physiological functions, including the protection of intestinal mucosal barrier and anti-inflammatory and antitumor effects.^61^ Smoking causes inflammation in the gut and decreases the levels of anti-inflammatory probiotics such as *Lactococcus*,^62^ whose abundance was also found to be higher in wastewater samples with lower population smoking rate. Other marker microorganisms such as *Granulicatella*, *Enterococcus*, and *Methanobrevibacter* are commonly found in the oral cavity and/or human gut.^63−65^*Granulicatella* as an opportunistic pathogen usually leads to infection in individuals with weakened immune systems.^66^

Previous studies have showed an association, either positive or negative, between Enterococcus and smoking.^67−69^ It is suggested as a biomarker for the low smoking rate in this study. Also interestingly, *Blautia* and *Methanobrevibacter*, microorganisms associated with gut imbalance, were also identified for low smoking rate, likely due to their roles as beneficial bacteria producing short-chain fatty acids.^42,70,71^*Ruthenibacterium* has been reported to produce butyrate,^72^ which has anti-inflammatory effects. *CAG-272* belongs to the Oscillospiraceae order, a gut microorganism that increases in Alzheimer’s patients.^73^

### 3.3 Verification of Microbial Biomarkers Using Nicotine Loading Rate

The nicotine concentrations in the wastewater samples were also analyzed and reported as daily nicotine loading rate (mg/1000 people/day) (Table S1). This nicotine loading rate reflects the total amount of smoking during the sampling days, while the population smoker percentage reflects the number of smokers usually with a long-term behavior of cigarette use. There is thus a likely agreement between nicotine loading rate and population smoking rate. A comparative correlation analysis was thus done between microbial relative abundance and the daily nicotine loading rate.

**Figure 4:**
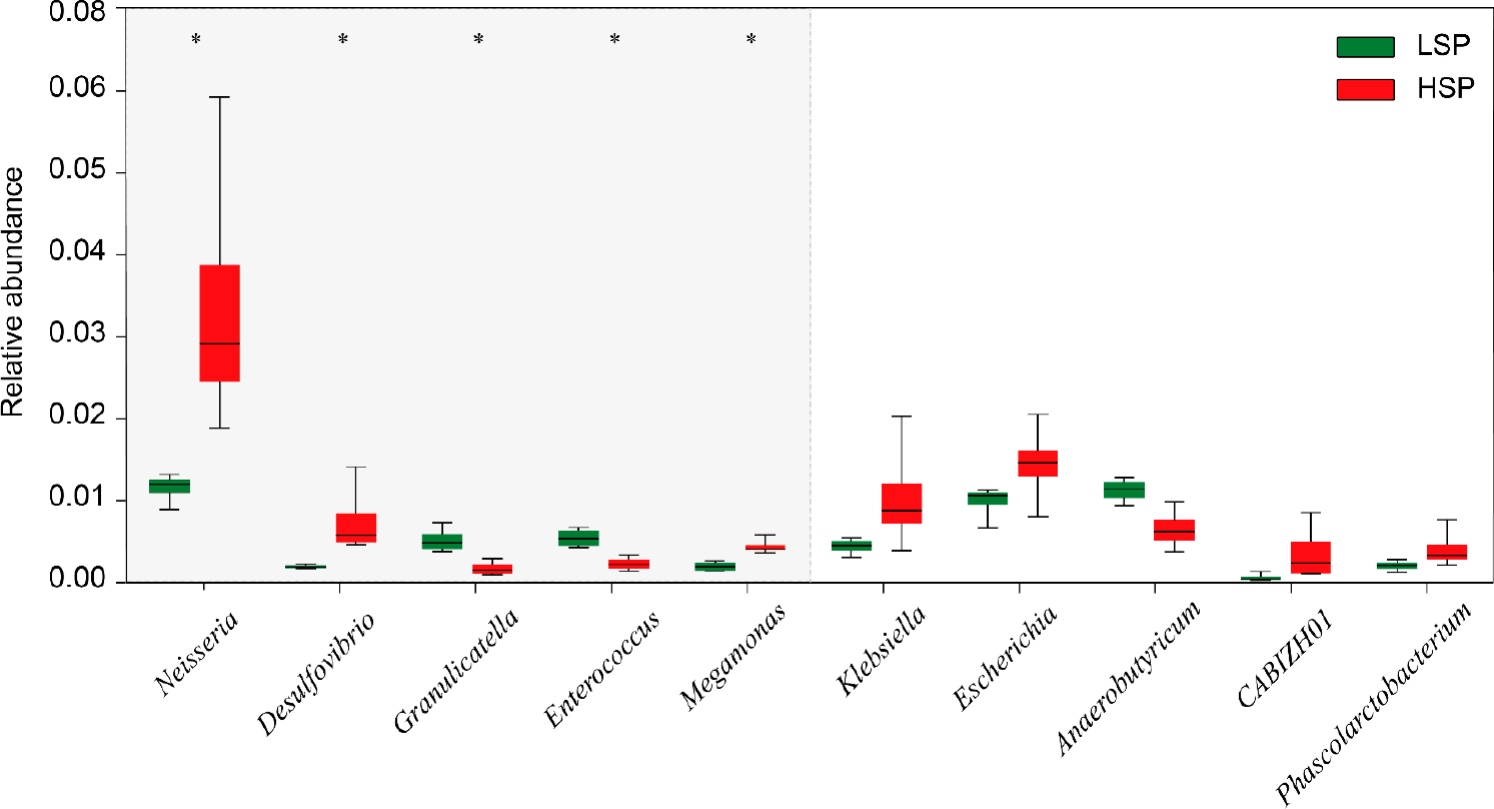
Relative abundance of the top 10 microorganisms identified based on metagenomic microbiome and daily nicotine loading rates. The 5 consensus microbial biomarkers are shaded, with significant differences of relative abundance (*p* < 0.05).

The marker microorganisms suggested by nicotine are similar to those reported in section 3.2. The top 10 microorganisms are primarily all derived from the Clostridia and Oscillospiraceae families (Figure S4). Among the marker microorganisms, *Neisseria*, *Desulfovibrio*, *Granulicatella*, *Enterococcus* and *Megamonas* showed significant differences between low and high smoking rates (Figure 4). Also, they are consensus microbial biomarkers that were suggested by both the wastewater concentration of nicotine and the LEfSe analysis on wastewater metagenomes.

### 3.4 Co-occurrence of Potential Biomarker Microorganisms in the Wastewater

**Figure 5:**
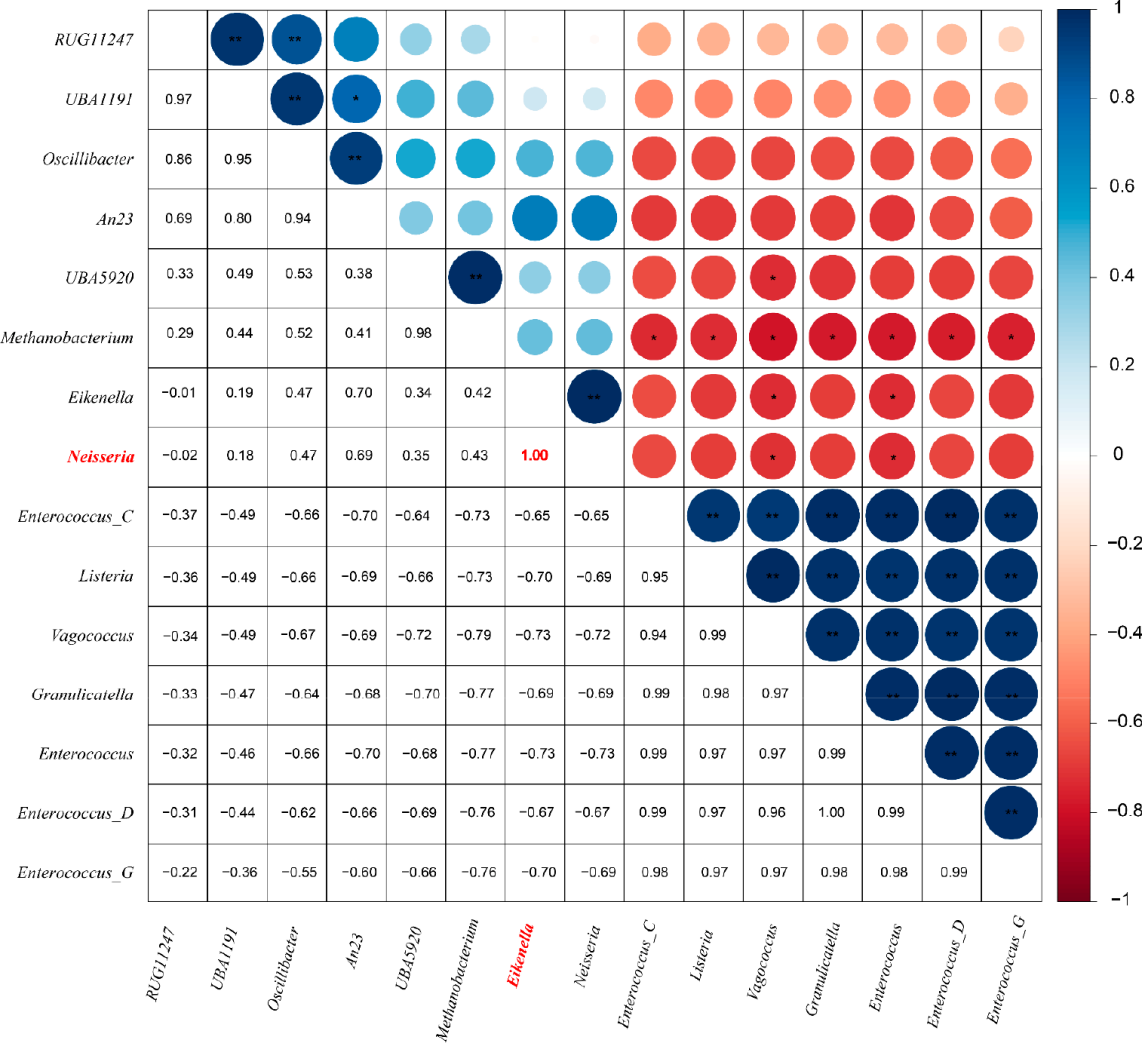
Heatmap showing the co-occurrence of the top 15 human-related genera in wastewater based on global Pearson correlations.

Two major clusters of microbial co-occurrence in wastewater, as shown in Figure S5, were profiled for their correlation with each other. One major cluster (including *Enterococcus_C*, *Enterococcus_D*, *Granulicatella*, *Listeria*, *Enterococcus*, *Vagococcus*, *Enterococcus_G* and *Enterococcus_B*) contains microorganisms that are commonly found in the human mouth and intestines and have a high chance of causing infections. Most of them have a high degree of co-occurrence with other microorganisms (Figure 5). *Granulicatella* and *Enterococcus* were also identified as biomarker microorganisms through LEfSe analysis. In addition, *Listeria* and *Vagococcus* are often found in smoked foods and can cross the intestinal barrier and the blood–brain barrier and cause bacteremia or meningitis.^74−76^

Another major cluster (*Haemophilus*, *Aggregatibacter*, *UMGS363*, *Mitsuokella*, *Helicobacter*, *Ruminococcus*, *CAG-603*, *SFGY01*, *UBA1394* and *RQCD01*) also includes pathogenic bacteria associated with the mouth and gut. Among them, *Haemophilus* was suggested as a biomarker microorganism by the LEfSe analysis. *Haemophilus* primarily colonizes the respiratory system and can lead to pneumonia.^77^*Helicobacter* is a prevalent pathogen known for its association with gastric cancer in the stomach.^78^*Aggregatibacter* is commonly found in the oral cavity and is considered a pathogenic bacterium.^79^ This is consistent with higher levels of bloodborne pathogens (e.g., *Neisseria*, *Haemophilus*) being detected in wastewater from WWTPs with high smoking rates. There is a high correlation between *Neisseria* and *Eikenella* (Figure 5). Overall, the network analysis revealed that the pathogenic bacteria clustered with each other, while the beneficial bacteria (such as short-chain fatty acids producers^80,81^) were scattered in the network, including *Oscillibacter*, *Caproiciproducens* and *Pseudobutyrivibrio*.

### 3.5 Association of Microbial Functions and Metabolisms to Smoking

**Figure 6:**
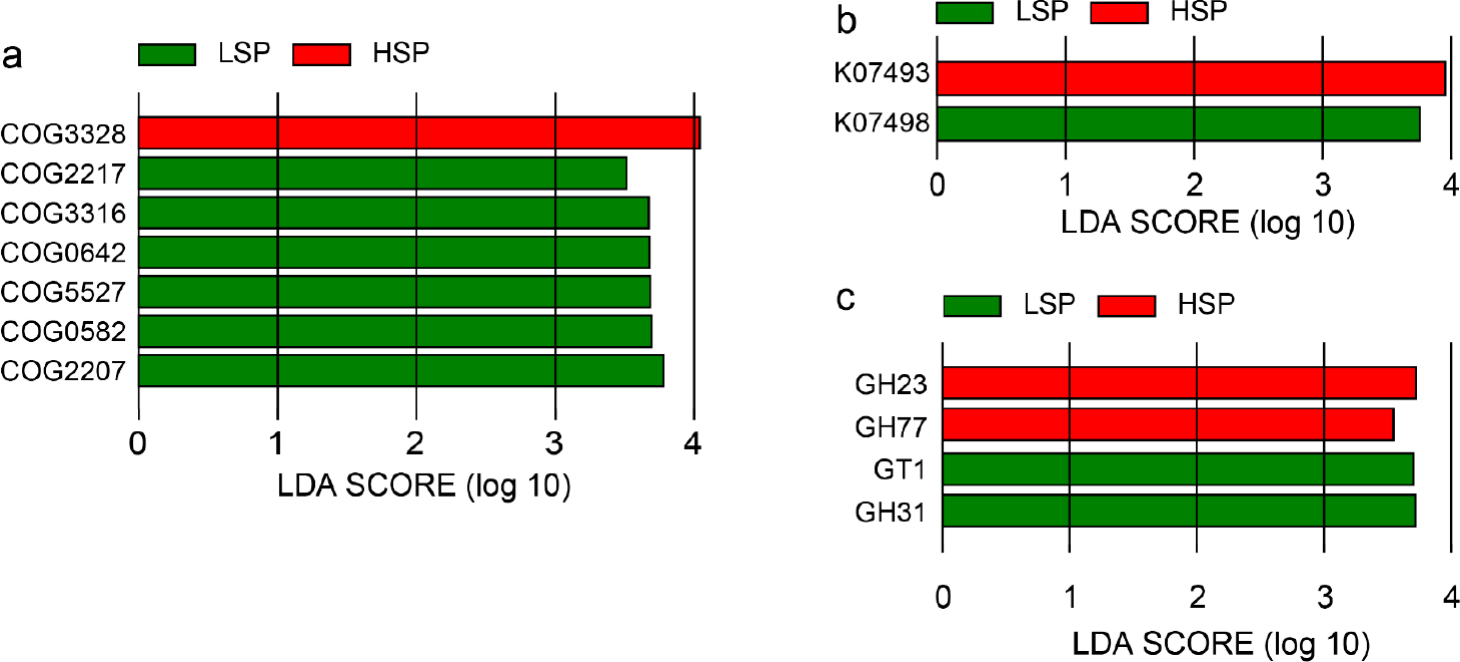
COGs functions and KEGG pathways of human gut microorganisms in wastewater metagenomes. (a) Seven COGs, (b) seven KOs, and (c) four CAZymes identified by LEfSe for smoking (*p* < 0.05, LDA ≥ 3.5).

Analysis of metabolic pathways suggests that the metabolic capacity for carbohydrates of human gut microbes in wastewater may be related to smoking rates. Figure 6 shows the COGs functions and KEGG pathways of human gut microorganisms in wastewater metagenomes, with more being listed in Tables S2, S3 and S4 (*p* < 0.05, LDA ≥ 3). LEfSe analysis at the COG category level showed significant differences in the J category (i.e., ribosomal structure and biogenesis) for the human gut derived microorganisms with regard to 25 categories in the COG database. A subset filtered by correlation was then analyzed using LEfSe, and 26 COGs were identified (Table S2). High smoking rate is mainly associated with transcription (K), antibiotic resistance (COG3328), nucleic acid metabolism (COG0317), and inorganic ion transport (COG0025) (Table S3). Wastewater microbiomes of low smoking rate, on the other hand, were characterized by COGs primarily involved in inorganic ion transport and metabolism (P) and mobilome: prophages, transposons (X).

The KEGG annotation of the wastewater metagenomes showed clear differences in the categories of Brite hierarchies, genetic information processing, and metabolism (Figure S6). Among 581 KEGG functional genes that were significantly associated with smoking rate, 31 distinguishing KOs (KEGG Orthology) were identified by LEfSe (Table S4). The associated KOs of HSP wastewater are mainly related to genetic information processing, such as K20276 involved in cell population sensing (QS) and K01467 involved in sensing changes in the external environment. The associated KOs of LSP wastewater were mainly related to the metabolism of carbohydrate, amino acid and energy (Table S3). These KOs expressed at distinguishable levels due to different smoking rates were previously reported to be involved in the pentose phosphate pathway, the manganese ion cell membrane transport, and a variety of cellular functions.^82^

Eight carbohydrases were discovered by LEfSe, which are GH23, GH31, GT1, GH77, GH43, GH51, PL8 and GT21 (Table S4). The effects of smoking on CAZy can be seen in the glycoside hydrolases due to the increased expression of GH23, a peptidoglycan lyase.^83^ This indicates that the cell membranes of associated microorganisms were likely damaged, and the cells were dividing. In comparison, GH31 (containing α-glucosidases) present in three screened representative microorganisms (*Blautia*, *Lactococcus* and *Enterococcus*) suggested that starch and cellulose metabolism is more active in microorganisms associated with the low smoking population.^84^ This observation is also verified by COG and KEGG. Among the 26 screened COGs, those associated with transcription, antibiotic resistance, and nucleic acid metabolism were predominantly linked to a high smoking rate, while the differentially represented KOs associated with a low smoking rate were primarily involved in carbohydrate, amino acid, and energy metabolism. These findings indicate that smoking significantly affects the intestinal barrier, cellular transport, and cellular messaging in the human gut.

### 3.6 Implications for Wastewater-Based Epidemiology of Smoking

The findings in this study suggest that the smoking-altered composition and functions of the microbial community in human guts can be detected after they are shed and collected in domestic wastewater. The human gut derived wastewater microbiomes were successfully screened for potential marker microorganisms of smoking rate at the population level. These biomarkers were also verified with previous studies conducted directly on human gut microbiomes.

Chemical markers of smoking have been employed for the consumption of cigarettes, e-cigarettes, or other ongoing nicotine replacement treatments over a short period of time. In comparison, the suggested microbial biomarkers in this study reflect the change in human gut microbiome due to the long-term smoking behavior. Although common microbial biomarkers were suggested by the nicotine daily loading rate (averaged over a week), the benefits of using microbial biomarkers are the high relevance to the impacts of smoking on human health, in terms of microbial abundance, functions, and metabolisms.

This study employed an approach of wastewater-based epidemiology, which has its own uncertainties associated with wastewater sampling, biomarker stability, sample analysis, and data analytics.^85^ Also, the variation of population smoking rate (which was extrapolated from 2 years of statistics) around 6% might have led to limited capacity of biomarker discovery in wastewater. In addition, the wastewater transportation in sewers may lead to changes of microbial community in sampled wastewater over a long hydraulic retention time.^86,87^ Further research on the decay of potential microbial biomarkers is essential to identifying a stable biomarker of smoking. Additionally, other human lifestyle factors, such as obesity and physical activity, can influence the composition of human gut microbiota.^88,89^ Similarly, microorganisms in wastewater can be influenced by factors such as the population size served by the treatment plant, in-sewer transport time, and local weather conditions.^90^ It is important to note that wastewater contains a significant number of nonfecal microorganisms, which can potentially interfere with the analysis.^91^ These confounding factors should be delineated in future studies. A future comprehensive study involving a large number of wastewater samples and other impacting environmental factors would be required to deliver a quantitative metric of the percentage of smokers in the population through wastewater-based epidemiology.

## Supporting Information Available

Alpha rarefaction curve of microbial richness in wastewater samples; most abundant phyla of the human gut microbial community detected in wastewater; top 10 microbial genera with the highest correlation between their relative abundance and the population smoking percentage; top 10 microbial genera with the strongest correlation between the daily nicotine loading rate and microbial abundance; co-occurrence network of human gut microbiome detected in wastewater; three KEGG pathways identified by LEfSe for smoking rates; details about sampled WWTPs of each catchment; representative COGs in sewage; representative KOs of human-related microorganisms in sewage from areas with different smoking rates; and representative CAZymes in sewage (PDF)
