# Supplementary material for: Associations between Wastewater Microbiome and Population Smoking Rate Identified Using Wastewater-Based Epidemiology
Source: Environ Health (Wash). 2023 Oct 19;1(6):394–404. doi: 10.1021/envhealth.3c00105 (PMC11504251; doi:10.1021/envhealth.3c00105)
Supplement: Supplementary file 1 — eh3c00105_si_001.pdf [file eh3c00105_si_001.pdf]

Supporting Information

Associations between wastewater microbiome and population smoking rate identified using wastewater-based epidemiology

*Jiangping Wu[a], Shuxin Zhang[a], Yan Chen[a], Jiawei Zhao[a], Tanjila Prosun[a], Jake William O'Brien[b], Jochen F. Mueller[b], Ben J. Tscharke[b], Lachlan Coin[c, d], Stephen P. Luby[e], Faisal I. Hai[a], Tanya Buchanan[f], Guangming Jiang[a, *]*

[a]School of Civil, Mining, Environmental and Architectural Engineering, University of Wollongong, Australia

[b]Queensland Alliance for Environmental Health Sciences (QAEHS), The University of Queensland, Brisbane, Queensland4102, Australia

[c]Department of Clinical Pathology, The University of Melbourne, Parkville, Melbourne, Australia

[d]Department of Microbiology and Immunology, The University of Melbourne, Parkville, Melbourne, Australia

[e]Division of Infectious Diseases and Geographic Medicine, Stanford University, Palo Alto, CA 94034

[f]Cancer Council Australia, Sydney, New South Wales, Australia

[*] Corresponding author. E-mail: gjiang@uow.edu.au; guangming.jiang@gmail.com.

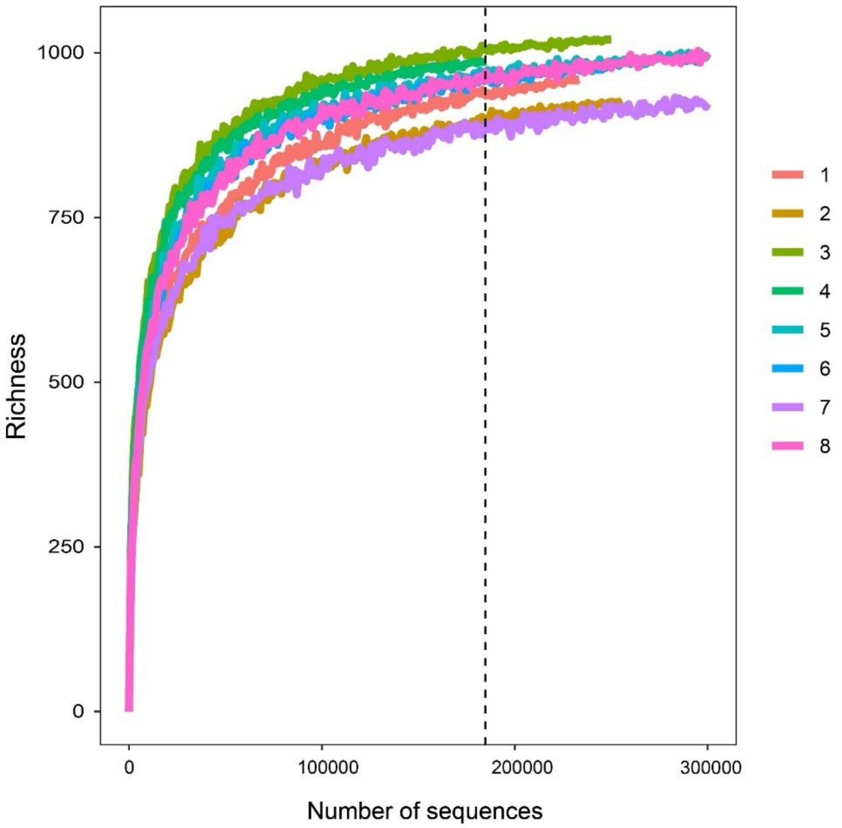

Figure S1. Alpha rarefaction curve of microbial richness in wastewater samples.

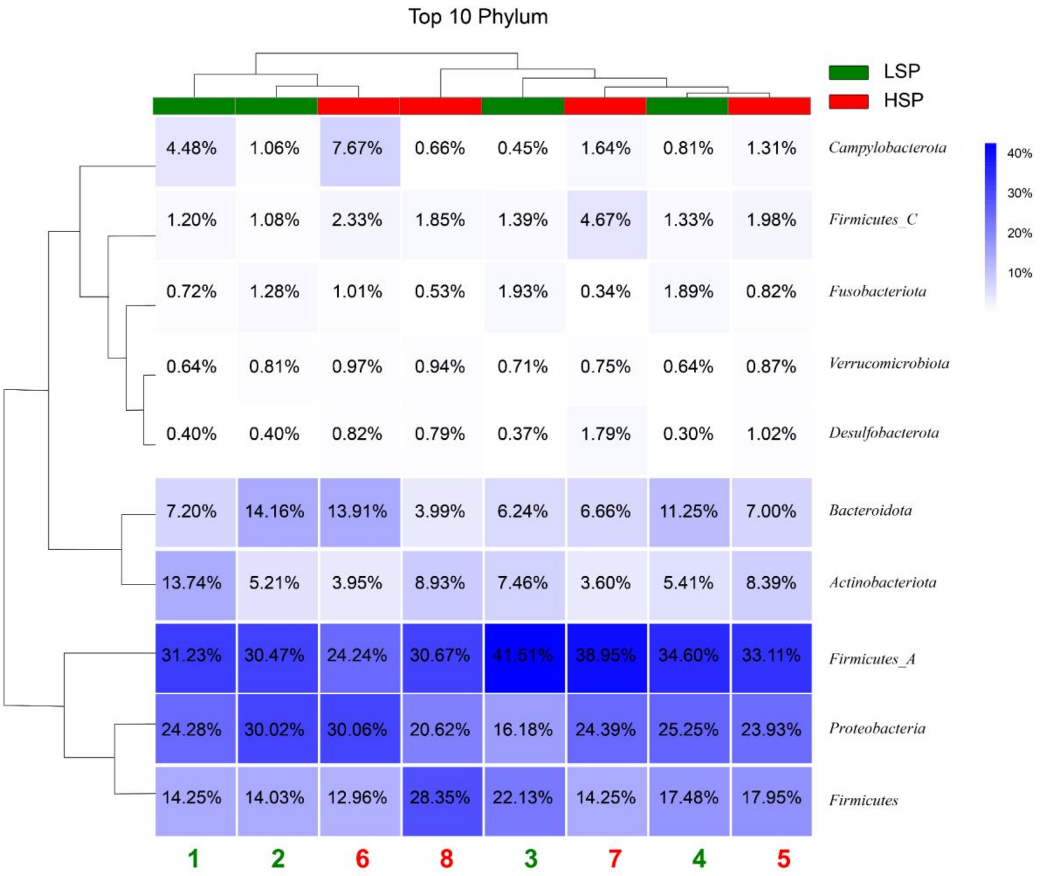

Figure S2. The 10 most abundant phyla of the human gut microbial community detected in wastewater.

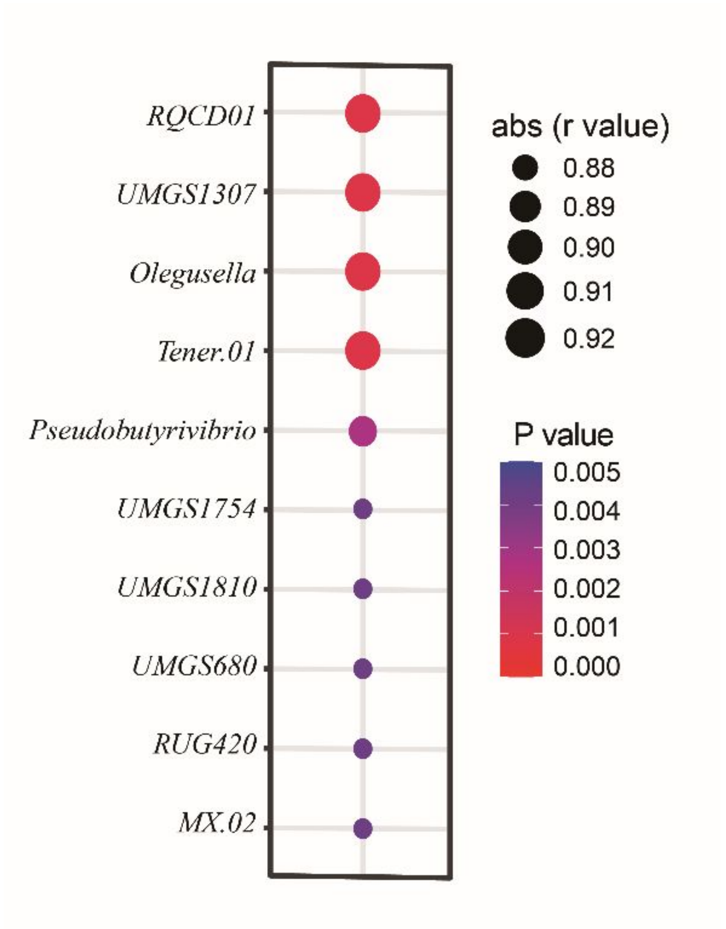

Figure S3. The top 10 microbial genera with the highest correlation (based on a Spearman's test, with a significance level of $p < 0.05$) between their relative abundance and the population smoking percentage.

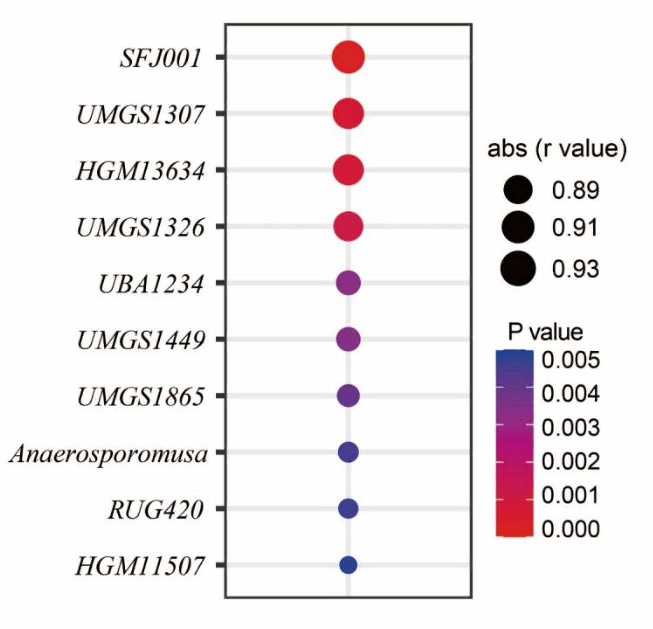

Figure S4. The top 10 microbial genera with the strongest correlation (based on a Pearson's test, with a significance level of $p < 0.05$) between the daily nicotine loading rate and microbial abundance.

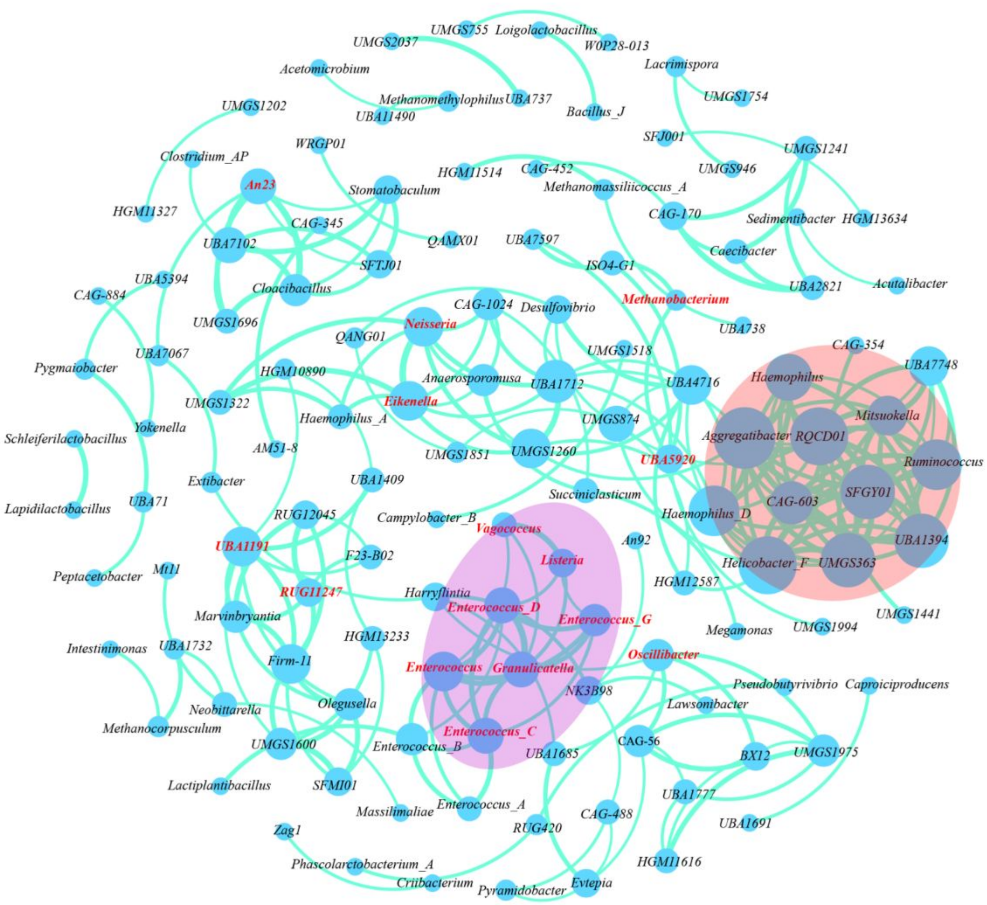

Figure S5. The cooccurrence network of human gut microbiome detected in wastewater ($p < 0.01$, $|r| > 0.6$). The thickness of connection lines indicates the correlation value; while the node size indicates the importance level. Top 15 human-related genera are highlighted in red.

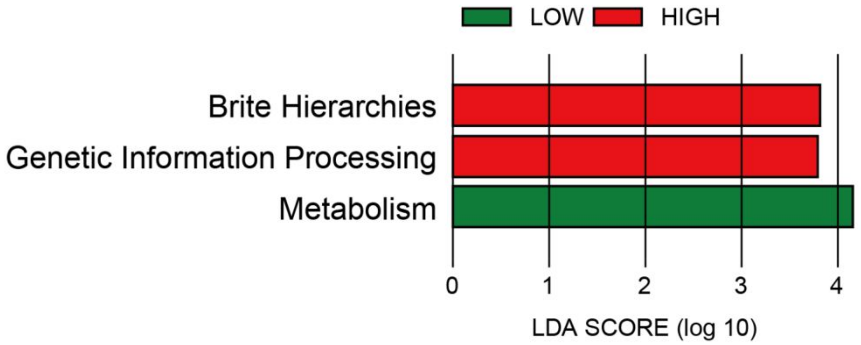

Figure S6. The 3 KEGG pathways (classification at level 1) identified by LEfSe for smoking rates. ($p < 0.05$, LDA ≥ 3.5).

Table S1. Sampled WWTPs and corresponding population smoking rate (smoker percentage and nicotine loading rate in wastewater) of each catchment.

WWTP	Smoker percentage 2017[*]	Smoker percentage 2014[*]	Average smoker percentage	Nicotine loading rate[†]	Flow (ML / day)	Population (Census 2016)	STATE	C/R
1	8.35%	11.31%	9.72%	716.90	<50	30,000 to 150,000	NSW	Capital
2	10.69%	13.70%	12.10%	1160.70	>50	>150,000	NSW	Capital
3	11.48%	11.52%	11.50%	1929.50	>300	>150,000	NSW	Capital
4	11.59%	10.75%	11.16%	1064.70	>100	>150,000	NSW	Capital
5	14.95%	15.75%	15.34%	2303.80	<50	30,000 to 150,000	QLD	Capital
6	15.40%	16.92%	16.14%	1736.10	<50	>150,000	QLD	Capital
7	17.21%	18.13%	17.66%	3618.70	>50	>150,000	QLD	Regional
8	17.79%	19.87%	18.80%	3615.30	<50	30,000 to 150,000	QLD	Regional

* Percentage of people aged 18 years and over who were current smokers.

† Nicotine daily loading rate (mg/1,000 people/day) (average across week)

Table S2. List of representative COGs of human-related microorganisms in sewage from areas with different smoking rates ($p < 0.05$, LDA ≥ 3).

No.	Accession number	Smoking rate	Category(L2)	Category(L1)	Description
1	COG3328	HSP	X: Mobilome: prophages, transposons	CELLULAR PROCESSES AND SIGNALING	Transposase (or an inactivated derivative)
2	COG0587	HSP	L: Replication, recombination and repair	INFORMATION STORAGE AND PROCESSING	DNA polymerase III, alpha subunit
3	COG0086	HSP	K: Transcription	INFORMATION STORAGE AND PROCESSING	DNA-directed RNA polymerase, beta' subunit/160 kD subunit
4	COG2804	HSP	NWU: Cell motility / Extracellular structures / Intracellular trafficking, secretion, and vesicular transport	CELLULAR PROCESSES AND SIGNALING	Type II secretory pathway ATPase GspE/PulE or T4P pilus assembly pathway ATPase PilB
5	COG0741	HSP	M: Cell wall/membrane/envelope biogenesis	CELLULAR PROCESSES AND SIGNALING	Soluble lytic murein transglycosylase or regulatory proteins (may contain LysM/invasin domain)
6	COG0317	HSP	KT: Transcription / Signal transduction mechanisms	INFORMATION STORAGE AND PROCESSING/CELLULAR PROCESSES AND SIGNALING	(p)ppGpp synthase/hydrolase, HD superfamily

7	COG3829	HSP	KT: Transcription / Signal transduction mechanisms	INFORMATION STORAGE AND PROCESSING/CELLULAR PROCESSES AND SIGNALING	RocR-type transcriptional regulator, contains PAS, AAA-type ATPase, and DNA-binding Fis domains
8	COG0025	HSP	P: Inorganic ion transport and metabolism	METABOLISM	NhaP-type Na+/H+ or K+/H+ antiporter
9	COG2207	LSP	K: Transcription	INFORMATION STORAGE AND PROCESSING	AraC-type DNA-binding domain and AraC-containing proteins
10	COG0582	LSP	LX: /Mobilome: prophages, transposons	INFORMATION STORAGE AND PROCESSING/CELLULAR PROCESSES AND SIGNALING	Integrase/recombinase, includes phage integrase
11	COG5527	LSP	X: Mobilome: prophages, transposons	CELLULAR PROCESSES AND SIGNALING	Protein involved in initiation of plasmid replication
12	COG0642	LSP	T: Signal transduction mechanisms	CELLULAR PROCESSES AND SIGNALING	Signal transduction histidine kinase
13	COG3316	LSP	X: Mobilome: prophages, transposons	CELLULAR PROCESSES AND SIGNALING	Transposase (or an inactivated derivative), DDE domain
14	COG2217	LSP	P: Inorganic ion transport and metabolism	METABOLISM	Cation-transporting P-type ATPase
15	COG1192	LSP	DN: Cell cycle control, cell division, chromosome partitioning / Cell motility	CELLULAR PROCESSES AND SIGNALING	ParA-like ATPase involved in chromosome/plasmid partitioning or cellulose biosynthesis protein

					BcsQ
16	COG0436	LSP	E: Amino acid transport and metabolism	METABOLISM	Aspartate/methionine/tyrosine aminotransferase
17	COG0673	LSP	R: General function prediction only	POORLY CHARACTERIZED	Predicted dehydrogenase
18	COG2148	LSP	M: Cell wall/membrane/envelope biogenesis	CELLULAR PROCESSES AND SIGNALING	Sugar transferase involved in LPS biosynthesis (colanic, teichoic acid)
19	COG2239	LSP	P: Inorganic ion transport and metabolism	METABOLISM	Mg/Co/Ni transporter MgtE (contains CBS domain)
20	COG1737	LSP	K: Transcription	INFORMATION STORAGE AND PROCESSING	DNA-binding transcriptional regulator, MurR/RpiR family, contains HTH and SIS domains
21	COG1376	LSP	M: Cell wall/membrane/envelope biogenesis	CELLULAR PROCESSES AND SIGNALING	Lipoprotein-anchoring transpeptidase ErfK/SrfK
22	COG0695	LSP	O: Posttranslational modification, protein turnover, chaperones	CELLULAR PROCESSES AND SIGNALING	Glutaredoxin
23	COG0281	LSP	C: Energy production and conversion	METABOLISM	Malic enzyme
24	COG0855	LSP	P: Inorganic ion transport and metabolism	METABOLISM	Polyphosphate kinase
25	COG4815	LSP	S: Function unknown	POORLY CHARACTERIZED	Uncharacterized conserved protein

26	COG0569	LSP	TP: Signal transduction mechanisms/ Inorganic ion transport and metabolism	CELLULAR PROCESSES AND SIGNALING/METABOLISM	Trk/Ktr K+ transport system regulatory component TrkA/KtrA/KtrC, RCK domain

Table S3. List of representative KOs of human-related microorganisms in sewage from areas with different smoking rates ($p < 0.05$, LDA ≥ 3).

No.	Accession number	Smoking rate	Category(L2)	Category(L1)	Description
1	K07493	HSP	Not Included in Pathway or Brite	Unclassified: genetic information processing	putative transposase
2	K20276	HSP	Cellular Processes	Cellular community - prokaryotes	bapA; large repetitive protein
3	K02337	HSP	Genetic Information Processing	Replication and repair	dnaE; DNA polymerase III subunit alpha [EC:2.7.7.7]
4	K03060	HSP	Genetic Information Processing	Transcription	rpoZ; DNA-directed RNA polymerase subunit omega [EC:2.7.7.6]
5	K08309	HSP	Brite Hierarchies	Protein families: metabolism	slt; soluble lytic murein transglycosylase [EC:4.2.2.-]
6	K12267	HSP	Not Included in Pathway or Brite	Unclassified: metabolism	msrAB; peptide methionine sulfoxide reductase msrA/msrB [EC:1.8.4.11 1.8.4.12]
7	K01467	HSP	Environmental Information Processing	Signal transduction	ampC; beta-lactamase class C [EC:3.5.2.6]
8	K03750	HSP	Metabolism	Metabolism of cofactors and vitamins	moeA; molybdopterin molybdotransferase [EC:2.10.1.1]

9	K07741	HSP	Not Included in Pathway or Brite	Unclassified: genetic information processing	antB; anti-repressor protein
10	K07498	LSP	Not Included in Pathway or Brite	Unclassified: genetic information processing	putative transposase
11	K03776	LSP	Environmental Information Processing	Signal transduction	aer; aerotaxis receptor
12	K01533	LSP	Not Included in Pathway or Brite	Unclassified: metabolism	copB; P-type Cu2+ transporter [EC:7.2.2.9]
13	K17686	LSP	Environmental Information Processing	Signal transduction	copA, ctpA, ATP7; P-type Cu+ transporter [EC:7.2.2.8]
14	K06941	LSP	Brite Hierarchies	Protein families: genetic information processing	rlmN; 23S rRNA (adenine2503-C2)-methyltransferase [EC:2.1.1.192]
15	K00937	LSP	Metabolism	Energy metabolism	ppk1; polyphosphate kinase [EC:2.7.4.1]
16	K05875	LSP	Environmental Information Processing	Signal transduction	tar; methyl-accepting chemotaxis protein II, aspartate sensor receptor
17	K06213	LSP	Brite Hierarchies	Protein families: signaling and cellular processes	mgtE; magnesium transporter
18	K01740	LSP	Metabolism	Amino acid metabolism	metY; O-acetylhomoserine (thiol)-lyase [EC:2.5.1.49]
19	K00284	LSP	Metabolism	Carbohydrate	GLU, gltS; glutamate synthase

				metabolism	(ferredoxin) [EC:1.4.7.1]
20	K01182	LSP	Metabolism	Carbohydrate metabolism	IMA, malL; oligo-1,6-glucosidase [EC:3.2.1.10]
21	K07494	LSP	Not Included in Pathway or Brite	Unclassified: genetic information processing	putative transposase
22	K01784	LSP	Metabolism	Carbohydrate metabolism	galE, GALE; UDP-glucose 4-epimerase [EC:5.1.3.2]
23	K01915	LSP	Metabolism	Carbohydrate metabolism	glnA, GLUL; glutamine synthetase [EC:6.3.1.2]
24	K00864	LSP	Metabolism	Lipid metabolism	glpK, GK; glycerol kinase [EC:2.7.1.30]
25	K07480	LSP	Not Included in Pathway or Brite	Unclassified: genetic information processing	insB; insertion element IS1 protein InsB
26	K03499	LSP	Brite Hierarchies	Protein families: signaling and cellular processes	trkA, ktrA, ktrC; trk/ktr system potassium uptake protein
27	K01665	LSP	Metabolism	Metabolism of cofactors and vitamins	pabB; para-aminobenzoate synthetase component I [EC:2.6.1.85]
28	K00013	LSP	Metabolism	Amino acid metabolism	hisD; histidinol dehydrogenase [EC:1.1.1.23]
29	K06926	LSP	Not Included in Pathway or Brite	Poorly characterized	uncharacterized protein

30	K01673	LSP	Metabolism	Energy metabolism	cynT, can; carbonic anhydrase [EC:4.2.1.1]
31	K00784	LSP	Brite Hierarchies	Protein families: genetic information processing	rnz; ribonuclease Z [EC:3.1.26.11]

Table S4. List of representative CAZymes of human-related microorganisms in sewage from areas with different smoking rates ($p < 0.05$, LDA ≥ 3).

CAZy	Smoking rate	Full name	Mechanism
GH23	HSP	Glycoside Hydrolase Family 23	Unknown e
GH31	LSP	Glycoside Hydrolase Family 31	Retaining a
GT1	LSP	GlycosylTransferase Family 1	Inverting
GH77	HSP	Glycoside Hydrolase Family 77	Retaining a
GH43	LSP	Glycoside Hydrolase Family 43	Inverting e
GH51	LSP	Glycoside Hydrolase Family 51	Retaining e
PL8	LSP	Polysaccharide Lyase Family 8	b-elimination
GT21	LSP	GlycosylTransferase Family 21	Inverting
